# A unique phytochrome B gene in *Cuscuta campestris* and its responses during the initial stage of haustorium formation

**DOI:** 10.1007/s10265-025-01653-5

**Published:** 2025-06-24

**Authors:** Toshiya Yokoyama, Mariko Asaoka, Kazuhiko Nishitani

**Affiliations:** Department of Science, Faculty of Science, Kanagawa University, 3-27-1 Rokkakubashi, Kanagawa-Ku, Yokohama, Kanagawa 221-8686 Japan

**Keywords:** *Cuscuta*, Haustorium, Light, Parasitism, Phytochrome

## Abstract

*Cuscuta*, a genus of stem-parasitic plants, requires specific light and touch stimuli for host recognition and haustorium formation. Blue and far-red light promote parasitism, while red light inhibits it, but the roles played by light-responsive genes in the early stages of *Cuscuta* parasitism remain largely unexplored. This study utilized an in vitro* C. campestris* haustorium induction system to investigate this issue. RNA-sequence analysis revealed a group of differentially expressed genes that responded to the initiation of haustorium formation. Genes associated with light-signaling pathways, including phytochromes (*PHYs*), phytochrome-interacting factors (*PIFs*), and homeodomain-leucine zipper I/II transcription factors, were specifically up or downregulated in plants exposed to light for 4 h. Phylogenetic analysis demonstrated that two distinct forms of the *PHYB* gene, *CcPHYB1* and *CcPHYB2*, were present in all sequenced *Cuscuta* species. *CcPHYB2* was not expressed under normal light conditions, but was specifically upregulated under light regimes inducing haustorium formation; by contrast, *CcPHYB1* exhibited non-light-dependent expression. Furthermore, the induction of haustorium formation by far-red light was canceled by red light exposure but could be re-activated by subsequent re-exposure to far-red light. The levels of expression of *CcPHYB2*, *CcPIF1*, *CcPIF4*, *CcHOMEOBOX FROM ARABIDOPSIS THALIANA 3* (*CcHAT3*), *CcHOMEOBOX 2* (*CcHB2)* and *CcHB13* varied under different haustorium-inducing light conditions. Expression of these genes altered significantly upon exposure to blue and far-red light, but these changes were reversed in plants exposed to red light. These findings highlight the pivotal role of light-responsive signaling during the early stages of parasitism, providing valuable insights into the molecular mechanisms and evolution of light-mediated host recognition and parasitism in *Cuscuta* species.

**Supplementary Information:**

The online version contains supplementary material available at 10.1007/s10265-025-01653-5.

## Introduction

The genus *Cuscuta* contains more than 200 species of stem and leaf parasitic plants that lack functional roots and leaves. Most species have little or no photosynthetic capacity. Instead, they produce yellow thread-like shoots that coil around other angiosperms and obtain water and nutrients via haustoria (Dawson et al. 1994). *Cuscuta* plants are thus obligate parasites that can only survive on their own for 2–3 weeks, which means rapid host detection and haustorium formation are essential for their survival (Benvenuti et al. 2005; Dawson et al. 1994; Pan et al. 2022). Light is an important environmental cue used by *Cuscuta* during the parasitic process. Since plant photosynthetic tissues absorb red light but transmit and reflect far-red light, the ratio of red to far-red light (R/FR ratio) indicates the presence of potential host plants. Light stimuli play significant roles in guiding host-seeking behaviors, coiling, and haustorium formation in *Cuscuta* (Benvenuti et al. 2005; Lane and Kasperbauer 1965; Orr et al. 1996; Smith et al. 2021). Far-red light facilitates these parasitic behaviors, whereas red light has an inhibitory effect (Lane and Kasperbauer 1965; Tada et al. 1996); blue light also induces parasitic behavior (Haidar et al. 1997, 1998; Haidar 2003; Haidar and Boss 2009). These findings suggest that a complex relationship between phytochrome and cryptochrome signaling pathways underlies parasitic behavior, although the molecular mechanisms regulating the interplay between photoresponses and parasitism remain largely unknown.

The molecular mechanisms controlling formation of the haustorium and its intrusion into host tissue have been studied intensively in recent years. Studies in *C. campestris* indicate that a putative ortholog of *LATERAL ORGAN BOUNDARIES DOMAIN* (*CcLBD*), which is involved in auxin signaling and lateral root development in Arabidopsis (*Arabidopsis thaliana*) (Du and Scheres 2018), plays an important role in the formation of vascular connections between the haustorium and the host (Jhu et al. 2021). In addition, genes encoding homeodomain-leucine zipper (HD-ZIP) proteins, pectin methylesterase inhibitors (PMEIs), and ethylene-responsive element binding factors (ERFs), proteins acting in auxin and cytokinin signaling pathways, and cell wall-loosening enzymes act in haustorium formation in *C. campestris* or *C. chinensis* (Jhu et al. 2022). Elongation and endoreduplication of the haustorium apical cell were suppressed during invasion of Arabidopsis octuple *1-aminocyclopropane-1-carboxylic acid synthase (acs)* mutants by searching hyphae of *C. campestris*, suggesting that ethylene is also involved in *Cuscuta* parasitism (Narukawa et al. 2021).

Despite such insights into the process of visible haustorium formation, the molecular events that occur between the point when *Cuscuta* detects the specific light signals that induce parasitic behavior and the invasion of the host, *i.e*., those determining the onset of haustorium formation itself, are not yet understood. Haustorium formation in *Cuscuta* can be triggered by exposure to far-red light for 1 or 2 h, even when plants are subsequently transferred to dark conditions (Bawin et al. 2022, 2024; Jhu et al. 2021; Olsen et al. 2016; Tada et al. 1996). This indicates that a transfer to darkness does not reset the commitment to parasitic behavior induced by brief far-red light exposure, suggesting that the onset of parasitism occurs irreversibly within the first few hours after light exposure. Furthermore, when *C. japonica* plants were exposed alternately to far-red and red light every hour, only those exposed to far-red light last became parasitic, not those whose final exposure was to red light (Tada et al. 1996). Thus, although the parasitic process is induced by a short period of light irradiation, the process can still be canceled by changes in light quality; this plasticity of response is an important step that determines whether parasitism will be induced or canceled in a variable environment (Tada et al. 1996). The light-dependent initiation of haustorium formation is therefore a critical part of successful parasitism.

It is extremely difficult to determine the precise time of haustorium formation experimentally, given that haustorium formation can be initiated by host recognition (contact stimulus) as well as by light stimuli. *Cuscuta* may initiate parasite behavior without a living host plant as, given appropriate light and contact cues, they will attempt to parasitize artificial hosts such as acrylic rods or fibrous sticks (Bernal-Galeano et al. 2022; Tada et al. 1996). We therefore developed an in vitro haustorium induction system for *Cuscuta* (Kaga et al. 2020; Yokoyama et al. 2022, 2023), which enabled the reproducible induction of haustorium formation by controlling contact stimuli (glass slides) and allowed us to observe the onset of parasitism. In this study, we used this in vitro haustorium induction system to identify the light-regulated initiation stage of haustorium formation in *C. campestris*. In addition, to identify and investigate genes involved in haustorium induction, we conducted transcriptome analysis under three light conditions (blue and far-red light, which induce haustorium formation, and red light, which inhibits it) at two different growth stages (seedlings and mature shoots) using RNA-sequence analysis (RNA-seq). This analysis identified a set of genes whose expression was regulated during the light-regulated haustorium induction stage, enabling us to find the *C. campestris* orthologs of genes encoding photoresponsive proteins, including phytochromes (PHYs), phytochrome-interacting factors (PIFs) and homeodomain-leucine zippers (HD-ZIPs). Furthermore, analyses of both gene phylogeny and expression patterns during haustorium formation uncovered a novel phytochrome unique to *Cuscuta*, as well as its role in initiating parasitism.

## Materials and methods

### Plant materials and parasitic conditions

Seeds from pure lines of *Cuscuta campestris* Yuncker were collected from laboratory cultivars that had been self-pollinated at least seven times (Kaga et al. 2020). To induce germination, *C. campestris* seeds were soaked in concentrated sulfuric acid for 25 min to scarify and remove the seed coat, rinsed five times in pure water, and sown in Petri dishes on filter paper soaked in pure water. *C. campestris* seedlings were incubated under continuous white light (45 µmol m^−2^ s^−1^, PF40-S18WT8-D; Nippon Medical & Chemical Instruments Co., Ltd.) in a growth chamber at 25 °C.

The Columbia (Col-0) accession of Arabidopsis (*Arabidopsis thaliana* (L.) Heynh,) was used as the host for *C. campestris*. Arabidopsis seeds were sown on rockwool blocks (Rockwool B. V.) soaked in culture solution diluted 1000-fold with Hyponex (HYPONeX Japan Corp.) and incubated under white light (Light 16 h: Dark 8 h, 45 µmol m^−2^ s^−1^, PF40-S18WT8-D; Nippon Medical & Chemical Instruments Co., Ltd.) in a growth chamber at 22 °C. Plants grown for 6 to 7 weeks and to greater than 100 mm in height were used as hosts.

To induce parasitism, *C. campestris* seedlings greater than 30-mm long were attached to the stem of a host plant with surgical tape (Micropore™ Surgical Tape; 3M Company). Sets of parasites and hosts were incubated under blue light (450 nm, 9 µmol m^−2^ s^−1^, 3LH-64; Nippon Medical & Chemical Instruments Co., Ltd.) in a growth chamber at 25 °C for 2 d. Plants were then transferred to continuous white light conditions (45 µmol m^−2^ s^−1^, PF40-S18WT8-D; Nippon Medical & Chemical Instruments Co., Ltd.) in a growth chamber at 25 °C. After parasitism, the shoot was considered mature and used in subsequent experiments.

### In vitro haustorium induction system and light conditions

Seedlings of *C. campestris* greater than 30 mm in length (i.e., approximately 3 d after germination) or 30-mm long apical segments of mature shoots were used in all in vitro haustorium induction experiments. Haustoria were induced in vitro as previously described (Kaga et al. 2020; Yokoyama et al. 2023). Briefly, four *C. campestris* seedlings or mature shoots were placed on a 3% agar gel containing 0.1% Plant Preservation Mixture™ (Plant Cell Technology, Inc.) in a rectangular Petri dish (100 mm × 140 mm, 14.5 mm deep, Sterile No. 2 Square Schale; Eiken Chemical Co., Ltd.) and seven glass slides were placed on top of the seedlings as a tactile stimulus. Seedlings and shoots were incubated under blue (maximum wavelength 450 nm, 9 µmol m^−2^ s^−1^, 3LH-64; Nippon Medical & Chemical Instruments Co., Ltd.), red (maximum wavelength 660 nm, 9 µmol m^−2^ s^−1^, 3LH-64; Nippon Medical & Chemical Instruments Co., Ltd.) or far-red light (maximum wavelength 735 nm, 9 µmol m^−2^ s^−1^, ISL-1ISL-150X150-ff; CCS Inc.). Haustorium formation was observed using a stereomicroscope (Stemi305, Zeiss). Series of images showing haustorium formation were captured using a digital camera (WG-6, RICOH).

### RNA extraction

Approximately 10 mm of tissue section was cut from the apical portion of seedlings or mature shoots of *C. campestris* under a stereomicroscope after removing the apex and side shoots, and the tissue section samples were frozen in liquid nitrogen. Frozen tissues were crushed in a TissueLyser (TissueLyser2; QIAGEN). Total RNA was extracted using the RNeasy Plant Mini Kit (QIAGEN Inc.) with RNase-Free DNase (QIAGEN Inc.), according to the manufacturer's protocol.

### Transcriptomic analysis

RNA libraries, cyclized DNA, and DNA Nanoball (DNB) were prepared using the MGIEasy RNA Directional Library Prep Set (MGI Tech Co., Ltd.), the MGIEasy Circularization Kit (MGI Tech Co., Ltd.), and the DNBSEQ-G400RS High-throughput Sequencing Kit (MGI Tech Co., Ltd.), respectively, according to the manufacturer’s protocols. The DNBs were sequenced using a DNBSEQ-G400 sequencer (MGI Tech Co., Ltd.) at 2 × 100 bp. Low quality reads (quality score < 20 and/or < 40 bp) were removed using sickle software (ver. 1.33). High quality reads were mapped onto the reference genome (Vogel et al. 2018) using hisat2 (ver. 2.2.1). The mapped reads were counted by featureCounts (ver. 2.0.3) and the read counts were converted to transcripts per million (TPM) (Su et al. 2019). Analysis of differentially expressed genes (DEGs) was performed using the edgeR method in the TCC-GUI software package. Genes whose expression levels varied significantly (false discovery rate (FDR) < 0.01) under blue, red, and far-red light conditions, relative to dark conditions, were considered to be DEGs. The raw reads were deposited in the DNA Data Bank of Japan (DDBJ) Sequence Read Archive under BioProject PRJDB19976.

### Gene ontology (GO) enrichment analysis

Arabidopsis orthologs of *C. campestris* genes were identified by comparing the *C. campestris* protein sequences with those of Arabidopsis using protein Basic Local Alignment Search Tool (BLAST) (blastp) with the default settings on the National Center for Biotechnology Information (NCBI) website. The GO enrichment analysis was conducted using the shinyGO online tool (ver 0.80) (Ge et al. 2020) (https://bioinformatics.sdstate.edu/go).

### Phylogenetic analysis

Orthologs of the *C. campestris* protein were identified using blastp on the NCBI website by comparing *C. campestris* protein sequences with those of Arabidopsis. Protein sequences were obtained from the NCBI and plaBiPD databases (https://www.plabipd.de). Accessions and abbreviations are shown in Table S2. The phylogenetic analysis was performed using MEGA 11 (Tamura et al. 2021). Protein sequences were aligned using the MUSCLE method. Phylogenetic trees using amino acid sequences were constructed by the Maximum Likelihood algorithm and inferred from 1000 bootstrap replicates. The phylogenetic trees were visualized using MEGA 11 (Figs. 3a, b, 4a) and the iTOL online tool (https://itol.embl.de/) (Fig. 5b).

### Motif analysis

Conserved motifs present in PIF proteins were identified by using MEME (Bailey and Elkan 1994) with the zoops method. In the search option, the minimum and maximum widths were set to 6 and 50, respectively. The locations of the motifs in the protein sequences were visualized using TBtools (Chen et al. 2020).

### Quantification of gene expression by real-time PCR

RNA extracted from *C. campestris* explants was reverse transcribed in a SimpliAmp Thermal Cycler (Thermo Fisher Scientific, USA) using ReverTraAce qPCR RT Master Mix with gDNA remover (TOYOBO, Japan), according to the manufacturer's protocol. Primers used in qRT-PCR analyses are listed in Table S3. qRT-PCR analyses were performed using a QuantStudio 1 Real-Time PCR System (Thermo Fisher Scientific, USA) with SYBR™ Green qPCR Master Mix (Thermo Fisher Scientific, USA) and Power SYBR™ Green qPCR Master Mix (Thermo Fisher Scientific, USA). Each analysis contained three biological replicates, each with three technical replicates. Expression levels of each gene were determined using the relative standard curve method and normalized on expression of *CcTUBULIN* (Cc015968.t1 and Cc032054.t1) as an internal standard (Narukawa et al. 2021). The ⊿CT values in Fig. 6 were compared using a Student’s *t*-test in Microsoft Excel. Multiple comparison of the ⊿CT values in Fig. 7 were performed by a one-way analysis of variance, followed by Tukey’s honesty significant difference test using R statistical software (version 4.2.1).

## Results

### Determination of the duration of light sufficient for in vitro induction of *C. campestris haustoria*

The process of haustorium formation has been defined by several previous studies, with the first visible morphological changes (i.e., stem enlargement, meristem development) being known as the “early stage” (Jhu et al. 2021), the “swelling stage” (Bawin et al. 2022), or the “initiation phase” (Jhu and Sinha 2022). To investigate the molecular basis of the very early stages of parasitism in *C. campestris*, we first used the in vitro haustorium induction system to determine the minimum period of light required for haustorium formation. This system allows precise control of touch stimuli by weighting *C. campestris* with glass slides, which is essential for parasitism but difficult to control in plant-plant experiments. Using this system, we previously demonstrated that both seedlings and mature shoots (defined as the apical part of the shoot after parasitism) of *C. campestris* do not induce haustorium formation under continuous red light conditions, while they successfully induce haustorium formation under blue and far-red light conditions (Yokoyama et al. 2023). In this study, we therefore exposed *C. campestris* seedlings and mature shoots to blue or far-red light for periods of between 1 and 4 h, followed by dark incubation for a total of 48 h, after which we evaluated the rate of haustorium formation.

**Fig. 1:**
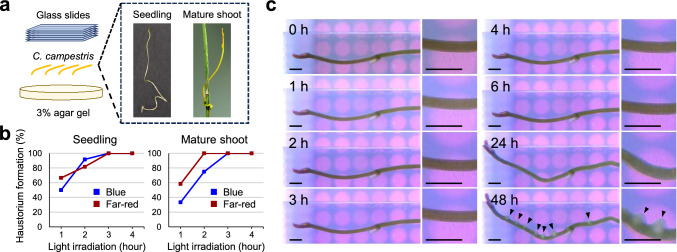
**a.** Diagram showing the in vitro haustorium induction system. Seedlings or mature shoots of *Cuscuta campestris* were placed on agar gel in a Petri dish and weighed down with glass slides. **b**. Rates of haustorium formation in *C. campestris* seedlings (left-hand panel) and mature shoots (right-hand panel) incubated using the in vitro haustorium induction system. Samples were exposed to blue or far-red light for 1, 2, 3, or 4 h, and then placed in the dark for 47, 46, 45, or 44 h, respectively: *n* = 11–22. Haustorium formation was assessed by observation using a stereomicroscope. **c.** Time-series images showing haustorium formation in mature shoots of *C. campestris* under far-red light. Representative images, captured after 0, 1, 2, 3, 4, 6, 24, and 48 h of far-red light exposure, were obtained from a timelapse recording. Scale bars: 1 mm

Blue light induced haustorium formation in 50% of seedling samples after 1 h, in 91.7% of seedlings after 2 h, and in 100% of seedlings after 3 to 4 h. Far-red light induced haustorium formation in 66.7% of seedlings after 1 h, in 81.8% after 2 h, and in 100% after 3 to 4 h (Fig. 1b). In mature shoots, blue light induced haustorium formation in 33.3% of samples after 1 h, in 75% after 2 h, and in 100% after 3 to 4 h. Far-red light induced haustorium formation in 58.3% of mature shoots after 1 h, and in 100% after 2 to 4 h (Fig. 1b). Obvious morphological changes indicative of haustorium formation were observed after 24 h of light exposure (Fig. 1c). These results suggested that, although visible morphological changes were not apparent after several hours of light exposure, the internal processes leading to haustorium formation may have been initiated within this time frame. We designated this period of the light-regulated process of haustorium formation (the period during which the commitment to haustorium formation occurred despite the absence of visible changes) the "induction stage".

### Profiling of gene expression during the light-regulated induction stage

To investigate gene expression during the light-regulated induction stage, we performed an RNA-seq analysis of *C. campestris* seedlings and/or mature shoots after a short period of incubation in the in vitro haustorium induction system. Seedlings and/or mature shoots were exposed to blue, red, or far-red light or to dark conditions for 4 h. Sequenced reads were mapped against the *C. campestris* genome. The accuracy of the RNA-seq results was verified by Principal Component Analysis (PCA) (Fig. S1). DEGs were identified as those genes that exhibited significantly different levels of expression under each light condition (blue, red, and far-red) compared with the dark condition (FDR < 0.01).

**Fig. 2:**
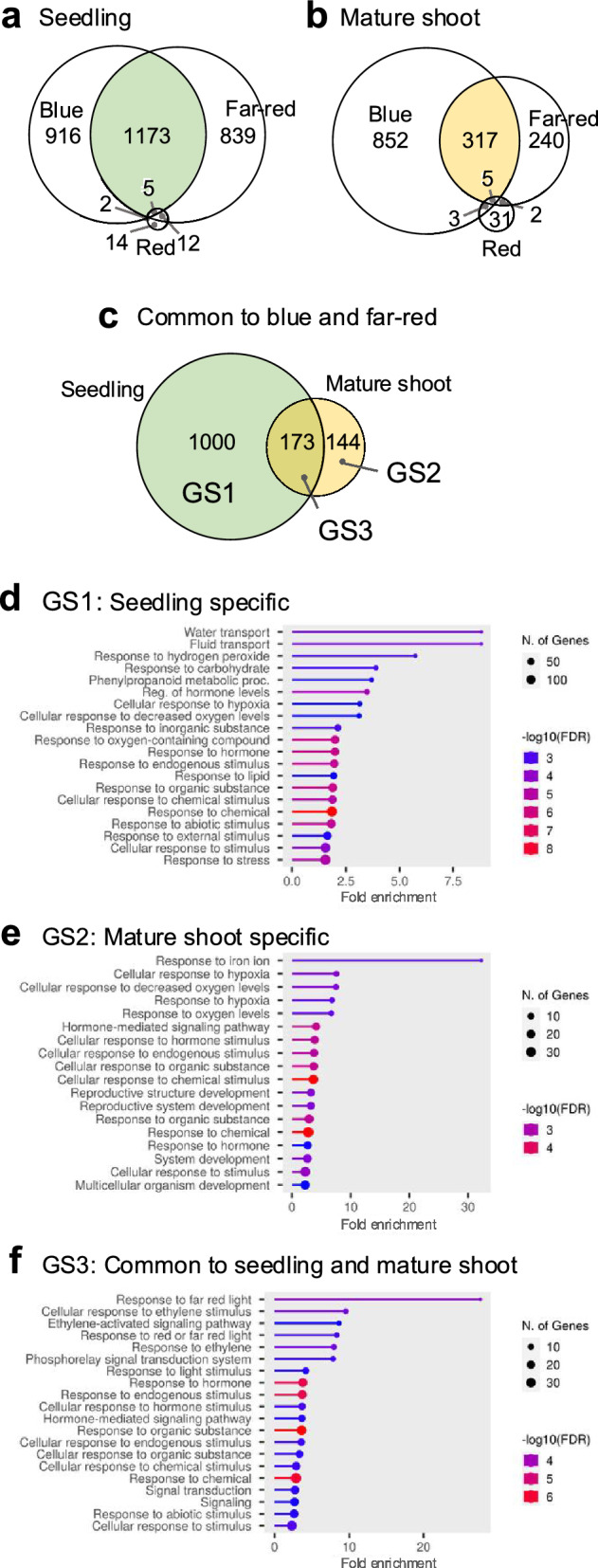
**a-b** Venn diagrams showing the numbers of DEGs identified by RNA-seq *versus* dark conditions in (**a**) seedlings and (**b**) mature shoots after 4 h exposure to blue (haustorium-inducing), far-red (haustorium-inducing), or red (non-haustorium-inducing) light. **c.** Venn diagram highlighting the overlap of DEGs common to blue and far-red light in seedlings and mature shoots. Seedling-specific DEGs were designated as Gene Set 1 (GS1), mature shoot-specific DEGs as Gene Set 2 (GS2), and DEGs common to both seedlings and mature shoots as Gene Set 3 (GS3). **d-f.** Gene Ontology (GO) enrichment analysis of (**d**) GS1, (**e**) GS2, and (**f**) GS3. The numbers of common genes and the false discovery rate (FDR) values of the top 20 terms with high fold enrichment are shown

For seedlings, 2096 genes (blue *vs.* dark), 2029 genes (far-red *vs*. dark) and 33 genes (red *vs*. dark) were identified as DEGs in each light condition. For mature shoots, 1177 genes (blue *vs*. dark), 564 genes (far-red *vs.* dark) and 41 genes (red *vs.* dark) were identified as DEGs in each light condition (Fig. 2a, b). The number of DEGs common to blue and far-red light, which both induced haustorium formation, but not to red light, which did not induce haustorium formation, was 1,173 in seedlings and 317 in mature shoots. Of the specific DEGs found in haustorium-inducing conditions, 1,000 were specific to seedlings, 144 to mature shoots, and 173 were common to both; these groups of DEGs were designated gene set 1 (GS1; seedling specific), gene set 2 (GS2; mature shoot specific), and gene set 3 (GS3; common to seedlings and mature shoots) (Fig. 2c).

Since few *C. campestris* genes have been characterized in genome databases, we identified the putative Arabidopsis orthologs of the *C. campestris* genes in each gene set (GS) using BLAST. We then performed a GO enrichment analysis for each GS using these Arabidopsis orthologs. The GO enrichment analysis of GS1 highlighted biological processes such as "water and fluid transport", "response to hydrogen peroxide", and "response to carbohydrate" (Fig. 2d). DEGs unique to mature shoots were, by contrast, enriched in processes related to "response to iron ion", "hypoxia", and "oxygen levels" (Fig. 2e). Genes in GS3, which contained DEGs common to seedlings and mature shoots, were, however, significantly enriched in GO terms associated with “response to far-red light” and “ethylene signaling” pathways (Fig. 2f). We considered it probable that such DEGs in GS3 were associated with the induction stage and thus likely to represent the central molecular mechanisms driving haustorium induction in *C. campestris*, implying the important role of light in this process. GS3 included DEGs encoding phytochrome, PIFs, and HD-ZIP II transcription factors, which are all important in plant photoresponses (Table S1). We therefore investigated the phylogeny of these genes, as well as analyzing their expression profiles during the induction stage in greater detail.

### Phylogeny and responses to light of the two phytochrome B genes present in the *C. campestris* genome

Many studies have hypothesized that phytochrome B contributes to *Cuscuta* parasitism (Bawin and Krause 2024; Furuhashi et al. 1995, 2021; Haidar et al. 1997, 1998; Lane and Kasperbauer 1965; Li et al. 2010; Orr et al. 1996; Tada et al. 1996; Yokoyama et al. 2023). Despite this body of work, it remains unresolved which phytochrome(s) is involved. We therefore performed a BLAST search against the *C. campestris* genome using the amino acid sequence of Arabidopsis PHYB (AT2G18790) to identify putative phytochromes. This identified 10 *C. campestris* genes that showed high homology to *PHYB* (E-values = 0.0); these genes were presumed to be phytochromes. Similarly, in the closely related species, *C. australis*, five potential phytochrome genes were identified.

To investigate the relationships between these genes, we constructed a phylogenetic tree using the phytochromes from *Cuscuta* and phytochromes A, B, C and E from other land plants. This tree classified one of the putative *C. australis* phytochrome genes (*RAL52740.1*) as *PHYA*, two (*RAL47423.1* and *RAL38571.1*) as *PHYB*, one (*RAL39348.1*) as *PHYC*, and one (*RAL50301.1*) as *PHYE*. Of the 10 putative phytochrome genes from *C. campestris*, two (*Cc004530.t1* and *Cc019681.t1*) were classified as *PHYA*, four (*Cc031752.t1*, *Cc031342.t1*, *Cc020059.t1* and *Cc044589.t1*) as *PHYB*, two (*Cc006928.t1* and *Cc007754.t1*) as *PHYC*, and the remaining two (*Cc017131.t1* and *Cc041797.t1*) as *PHYE*. The ten putative phytochrome genes from *C. campestris* formed five pairs of two genes, which likely reflected the whole-genome doubling event known to have occurred in this species (Neumann et al. 2021). Accordingly, we designated the *C. campestris* putative phytochrome genes as *CcPHYA*, *CcPHYB*, *CcPHYC* and *CcPHYE*. The duplicated genes in a pair were distinguished by the suffixes “a” and “b”, thus, for example *CcPHYAa* and *CcPHYAb*, with the gene designated “a” in each pair being phylogenetically closer to its *C. australis* ortholog than that designated “b”.

Plant phytochromes contain six domains, namely, the N-terminal Per/ARNT/Sim (nPAS) domain, the cGMP-specific phosphodiesterases/adenylyl cyclases/FhlA (GAF) domain, the phytochrome-specific (PHY) domain, the PAS1 and PAS2 domains, and the histidine kinase-related domain (Hughes and Winkler 2024). The protein structures predicted from the amino acid sequences of CcPHYB and CaPHYB contained all the expected phytochrome domains (Fig. S2).

**Fig. 3:**
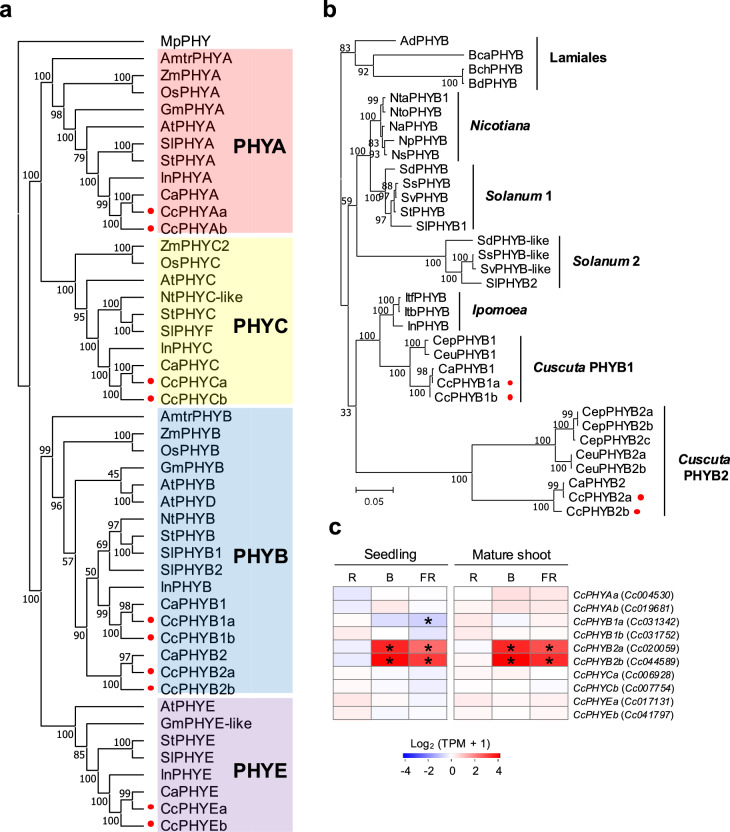
**a.** Phylogenetic tree of phytochromes from land plants. Abbreviations: *Marchantia polymorpha* (Mp), *Amborella trichopoda* (Amtr), *Zea mays* (Zm), *Oryza sativa* (Os), *Glycine max* (Gm), *Arabidopsis thaliana* (At), *Solanum lycopersicum* (Sl), *Solanum tuberosum* (St), *Ipomoea nil* (In), *Cuscuta australis* (Ca), and *Cuscuta campestris* (Cc). Phytochrome groups are categorized as Phytochrome A (PHYA), Phytochrome B (PHYB), Phytochrome C (PHYC), and Phytochrome E (PHYE). **b.** Phylogenetic tree of PHYB from members of the order Solanales. Abbreviation: *Nicotiana attenuate* (Na), *Nicotiana plumbaginifolia* (Np), *Nicotiana sylvestris* (Ns), *Nicotiana tabacum* (Nta), *Nicotiana tomentosiformis* (Nto), *Solanum dulcamara* (Sd), *Solanum stenotomum* (Ss), *Solanum verrucosum* (Sv), *Solanum tuberosum* (St), *Solanum lycopersicum* (Sl), *Ipomoea triloba* (Itb), *Ipomoea trifida* (Itf), *Ipomoea nil* (In), *Cuscuta europaea* (Ceu), *Cuscuta epithymum* (Cep), *Cuscuta australis* (Ca), and *Cuscuta campestris* (Cc). As an outgroup, PHYBs from members of the order Lamiales were included. Abbreviations: *Abeliophyllum distichum* (Ad), *Brandisia cauliflora* (Bca), *Brandisia chevalieri* (Bch), and *Brandisia discolor* (Bd). Red circles indicate CcPHY. **c.** Heatmap showing gene expression profiles (log₂ TPM + 1) of *CcPHY*. The color scale shows expression in seedlings and mature shoots under different light conditions, red (R), blue (B), and far-red (FR), relative to the corresponding levels in the dark. Asterisks indicate significant differences compared with the dark condition (FDR < 0.01)

We noted that the *CcPHYB* genes formed two distinct clades, one of which was closely related to the canonical *PHYB* found in other angiosperms, while the other had diverged within *C. campestris* and *C. australis* (Fig. 3a). We therefore categorized the *C. campestris PHYB* genes as *CcPHYB1* and *CcPHYB2*; further details are shown in Table S2. To examine the phylogeny of PHYB in *Cuscuta* in more detail, we identified PHYBs in two other *Cuscuta* species, *C. epithymum* and *C. europea*, and then created a phylogenetic tree using PHYB sequences from the order Solanales (Fig. 3b). This showed that the *Cuscuta* PHYBs fell into two distinct clades. All four *Cuscuta* species possessed both PHYB1 and PHYB2, which implies PHYB2 evolved in this genus. The long branches of *Cuscuta* PHYB2s in the phylogenetic tree suggested that these proteins contained a large number of amino acid changes.

RNA-seq identified *CcPHYB2* as one of the genes showing large changes in expression in seedlings and mature shoots under blue and far-red light (*vs.* dark, FDR < 0.01; categorized in GS3 in Fig. 1c); by contrast, *CcPHYB1* and the other *CcPHYs* showed weak or no significant changes in expression (Fig. 3c). Notably, the absolute level of expression of *CcPHYB2* was almost zero in the dark and under red light, but it was highly expressed under blue or far-red light (Fig. S3). Together, these results showed that *Cuscuta* possessed two distinct forms of PHYB, one of which was unique to this genus. The *Cuscuta-*specific form of *PHYB* was highly responsive to light during the induction stage, an expression pattern that differed from those of the other *PHY* genes.

### Characterization of PIFs in *Cuscuta*

**Fig. 4:**
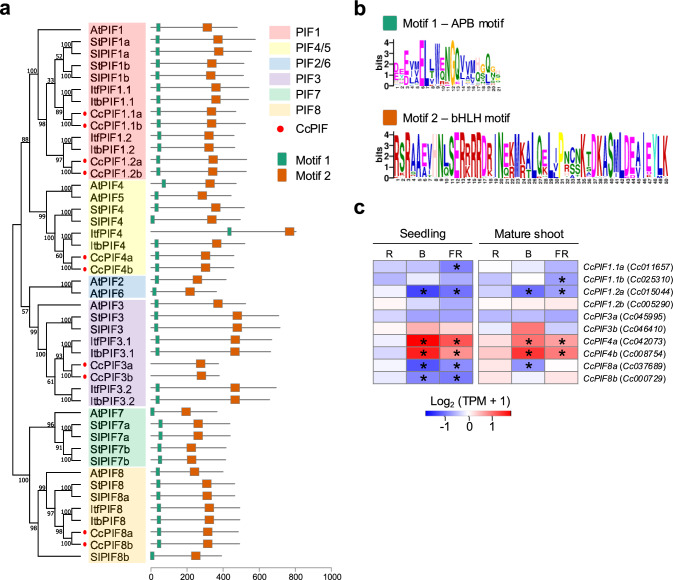
**a-b.** Phylogenetic tree of PIF and its conserved sequence. **a.** Phylogenetic tree of PIFs from *Arabidopsis thaliana* (At), *Solanum lycopersicum* (Sl), *Solanum tuberosum* (St), *Ipomoea triloba* (Itb), *Ipomoea trifida* (Itf) and *Cuscuta campestris* (Cc). Red dots indicate CcPIFs. The position of conserved motifs in the PIF proteins identified using MEME, motif-based sequence analysis tools, are shown by the green box (Motif 1: APB motif) and orange box (Motif 2: bHLH motif). **b.** The detailed sequence of the two conserved motifs in PIF proteins detected by using, MEME. Motifs were detected by inputting all of PIF protein sequence indicated in Fig. 4a. **c.** Heatmap showing the gene expression profiles (log₂ TPM + 1) of *CcPIFs*. The color scale shows expression in seedlings and mature shoots under different light conditions, red (R), blue (B), and far-red (FR), relative to the corresponding levels in the dark. Asterisks indicate significant differences compared with the dark condition (FDR < 0.01)

Next, we focused on *PIF* expression in *Cuscuta*, a member of the basic helix-loop-helix (bHLH) transcription factor family, which interacts with phytochromes. Although, genes encoding the bHLH family have been annotated by Vogel et al. (2018) and *PIF3* is conserved in the genus *Ipomoea* (Nie et al. 2023), the precise number and expression profiles of the *PIF* genes in *Cuscuta* species have not previously been determined. First, as in the case of searching *PIF* genes, we performed a BLAST search against the *C. campestris* genome using the amino acid sequence of Arabidopsis PIF3 to identify putative *CcPIFs.* Then, we found as many as 83 candidate genes (E-value < 0.05). To narrow down the potential *PIFs* in *C. campestris*, we performed a motif analysis of the *C. campestris* bHLH genes, identifying 8 genes containing the APB motif (a conserved sequence motif that mediates interaction with PHYB in PIF, Khanna et al. (2004)) that were annotated as putative *CcPIFs* (Fig. 4a, b). We found that the *C. campestris* bHLH gene with the highest similarity to both the Arabidopsis PIF3 and the *Ipomoea* PIF3 was not included among the above putative *CcPIFs*. Therefore, we made an exception and designated this highly homologous gene*,* along with its duplicated genes, as *CcPIF3*. In total, we identified 10 putative *PIF* orthologs in the *C. campestris* genome (Table S2). We subsequently conducted a phylogenetic analysis using *PIF* genes from Arabidopsis, members of the genera *Solanum* and *Ipomoea*, and *C. campestris*. This categorized the *PIF*s into six clusters (*PIF1*, *PIF2/6*, *PIF3*, *PIF4/5*, *PIF7*, and *PIF8*) based on the location of the *PIFs* from Arabidopsis (Fig. 4a; Table S2). The *PIF1* subfamily in *C. campestris* contained four genes, *Cc011657.t2* (*CcPIF1.1a*), *Cc025310.t1* (*CcPIF1.1b*), *Cc015044.t1* (*CcPIF1.2a*), and *Cc005290.t1* (*CcPIF1.2b*); the *PIF3* subfamily contained two genes, *Cc045995.t1* (*CcPIF3a*) and *Cc046410.t1* (*CcPIF3b*); the *PIF4* subfamily contained two genes, *Cc042073.t1* (*CcPIF4a*) and *Cc008754.t1* (*CcPIF4b*); and the *PIF8* subfamily contained two genes, *Cc037689.t1* (*CcPIF8a*) and *Cc000729.t1* (*CcPIF8b*). Of these *CcPIF* genes, *CcPIF1.2a*, *CcPIF4a*, and *CcPIF4b* showed a light-responsive pattern of expression during the induction stage (Figs. 4c, S3).

### Expression changes in specific *HD-ZIP I/II* genes in response to haustorium-inducing light signals

**Fig. 5:**
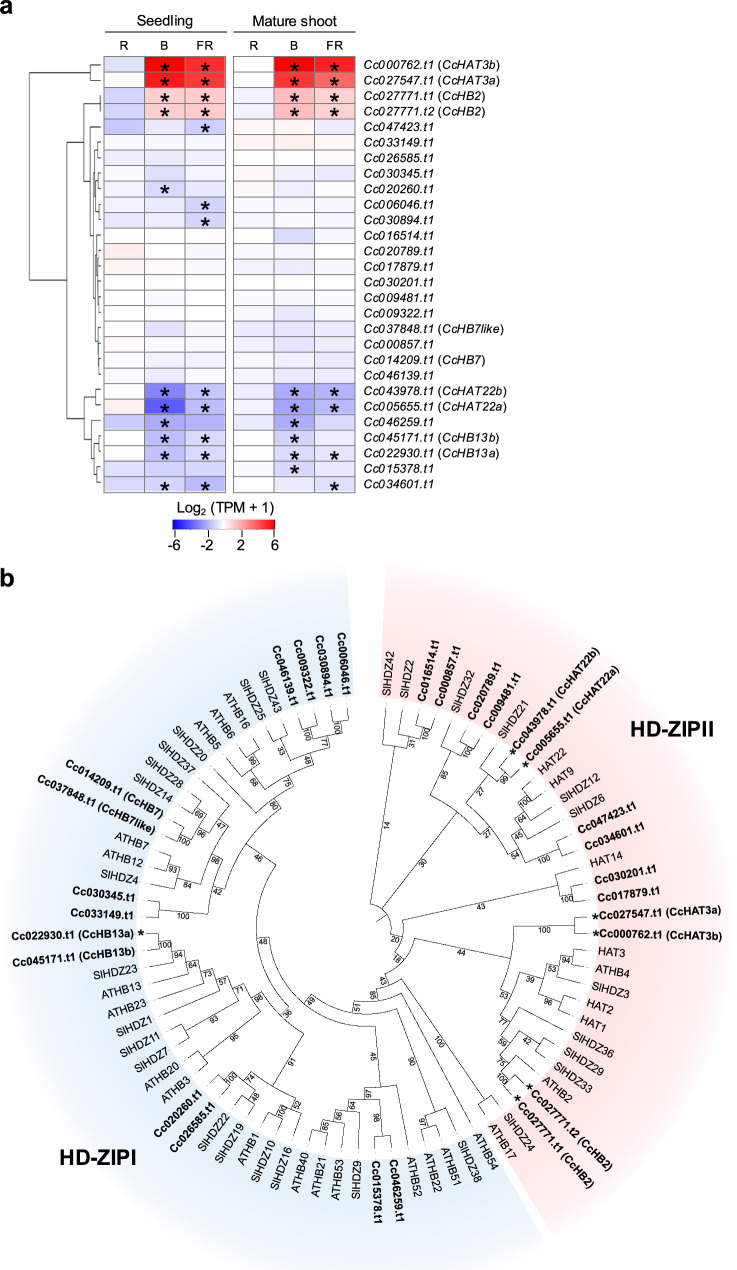
**a.** Heatmap showing gene expression profiles (log₂ TPM + 1) of *CcHD-ZIP I/IIs*. The color scale shows expression in seedlings and mature shoots under different light conditions, red (R), blue (B), and far-red (FR), relative to the corresponding levels in the dark. Asterisks indicate significant differences compared with dark conditions (FDR < 0.01). **b.** Phylogenetic tree of HD-ZIP I/IIs from *Arabidopsis thaliana, Solanum lycopersicum* (SlHDZ) and *Cuscuta campestris*. *CcHD-ZIP I/II* genes are shown in bold. Asterisks indicate DEGs

Previous studies revealed that HD-ZIP II proteins, which form a subfamily within the HD-ZIP family, play crucial roles in photoresponses, including shade avoidance, and meristem regulation (Ariel et al. 2007; Sessa et al. 2005). In *C. campestris*, two *HD-ZIP II* genes, *CcHOMEOBOX7* (*CcHB7*; *Cc014209.t1*) and *CcHB7like* (*Cc037848.t1*), are highly expressed, although expression of *CcHB7* fluctuates during the intrusion of the haustorium into host tissues (Jhu et al. 2022). Genes in the *C. campestris* genome that were annotated as *HD-ZIP I/II* in Vogel et al. (2018) were considered to be putative *CcHD-ZIP I/II* genes and subjected to further analysis. We identified seven putative *CcHD-ZIP I/II* genes as DEGs under haustorium-inducing conditions (Fig. 5a) and explored their relationships by constructing a phylogenetic tree containing these genes as well as *HD-ZIP I/II* genes from Arabidopsis and tomato (*Solanum lycopersicum*), a plant that is closely related to *Cuscuta* and whose *HD-ZIP* genes have been characterized (Hong et al. 2021) (Fig. 5b). Of the seven DEGs identified as putative *CcHD-ZIP I/II* genes, six were classified as *HD-ZIP II* genes, of which four, *Cc000762.t1*, *Cc027547.t1*, *Cc027771t1*, and *Cc027771t2*, were upregulated, and two, *Cc005655.t1* and *Cc043978.t1*, downregulated; the remaining gene, *Cc022930.t1*, which was downregulated, was classified as an *HD-ZIP I* gene (Figs. 5b, S3).

We used the results from this phylogenetic tree and of a BLAST analysis (Table S1) to name these *CcHD-ZIP I/II* genes as follows: *Cc000762.t1* and *Cc027547.t1* as *CcHOMEOBOX FROM ARABIDOPSIS THALIANA 3* (*CcHAT3*); *Cc027771* as *CcHOMEOBOX 2* (*CcHB2*); *Cc005655.t1* and *Cc043978.t1* as *CcHAT22*; and *Cc022930.t1* as *CcHB13*; full details are shown in Table S2. *CcHB7* (*Cc014209.t1*) was not identified as a DEG at the induction stage and showed little variation in expression (Figs. 5a, S3).

### Time-courses of *CcPHYB, CcPIF* and *CcHD-ZIP I/II* expression during the light-regulated induction stage

To gain a deeper understanding of the dynamics of gene expression during the light-dependent induction stage, we performed a qRT-PCR analysis on RNA samples extracted from seedlings and mature shoots grown in the in vitro haustorium induction system. We analyzed the expression of *CcPHYB1*, which was not identified as a DEG, and *CcPHYB2*, which was a DEG, as well as that of *CcPIF1.2*, *CcPIF4*, *CcHAT3*, *CcHB2*, and *CcHB13*. We were unable to prepare primers capable of analyzing *CcHAT22* (*Cc005655.t1* and *Cc043978.t1*) expression. In addition, we could not design primers capable of distinguishing between some pairs of duplicated genes with very similar sequences (*e.g.*, *CcPHYB1a* and *CcPHYB1b*); in such cases, we designed primers that targeted both members of a pair. Expression of *CcPHYB*, *CcPIF*, and *CcHD-ZIP I/II* in seedlings was examined under three conditions: (1) Constant dark for 24 h; (2) Blue light for 4 h, followed by dark for 20 h; and (3) Far-red light for 4 h, followed by dark for 20 h. We collected RNA samples at seven time-points: 0 (start point) and 1, 2, 3, 4, 6, and 24 h after the onset of light exposure.

**Fig. 6:**
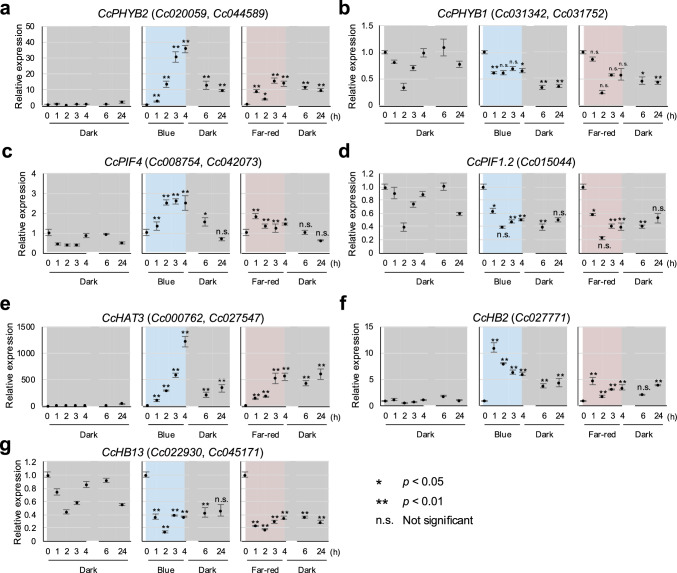
Expression of **a-b.** *CcPHYBs*, **c-d.** *CcPIFs*, and **e–g.** *CcHD-ZIP I/II* genes in the in vitro haustorium induction system under three different conditions. *Cuscuta campestris* was incubated in constant dark (left-hand panel), exposed to 4 h blue light followed by 20 h in the dark (center panel), or exposed to 4 h far-red light followed by 20 h in the dark (right-hand panel). Gene expression levels were assessed 1, 2, 3, 4, 6, and 24 h after the onset of light exposure and also at the start point (0 h). Expression of each gene was standardized on *C. campestris TUBULIN* (*CcTUB*, *Co032054.t1*) expression as an internal control and then relative to that at the start point (0 h). The value at 0 h was used for all three conditions. Data shown are ΔCt means ± SE. Asterisks indicate significant differences compared with the expression level in the dark at the same time point determined using Student’s *t*-test; *p < 0.05; **p < 0.01; n.s., not significant

The patterns of *CcPHYs*, *CcPIF* and *CcHD-ZIP I/II* expression obtained by qPCR were consistent with the RNA-seq analysis, which corresponded to 4 h of light exposure. The level of *CcPHYB2* (*Cc020059.t1*, *Cc044589.t1*) expression remained low in constant dark, while its expression increased in tissue exposed to blue and far-red light, peaking after 3 to 4 h, and then decreased in the dark over the next 20 h, although its level remained about tenfold higher at 24 h than the initial expression level (Fig. 6a). By contrast, *CcPHYB1* (*Cc031342.t1*, *Cc031752.t1*) expression showed moderate fluctuations under all three conditions and no clear light-dependent expression pattern was observed (Fig. 6b). *CcPIF4* (Cc008754.t1, Cc042073.t1) was highly expressed under both blue and far-red light; its expression decreased in the dark and became comparable to its expression at the start point or in constant dark by 24 h (Fig. 6c). By contrast, *CcPIF1.2a* (*Cc015044.t1*) was downregulated in blue and far-red light, and, by 24 h, its expression level was comparable to that at the start point or in constant dark (Fig. 6d). The expression of *CcHAT3* (*Cc000762.t1* and *Cc042073.t1*) was strongly upregulated in blue and far-red light, especially under blue light, with expression after 4 h was more than 1000-fold higher than at the start point or in constant dark. Its expression decreased after the transition from light to dark, but, after 24 h, it remained more than 100-fold higher than at the start point or under constant dark conditions (Fig. 6e). Expression of *CcHB2* (*Cc027771*) peaked after 1 h of light exposure and then decreased until the transition to darkness (Fig. 6f). *CcHB13* (*Cc022930.t1*, *Cc045171.t1*) expression was downregulated under both blue and far-red light and remained low after the transition to dark (Fig. 6g).

### Changes in *CcPHYBs, CcPIF* and *CcHD-ZIP I/II* expression were consistent with haustorium formation

To determine whether the observed changes of expression of *CcPHYBs, CcPIF*, and *CcHD-ZIP I/II* genes were associated with the initial process of haustorium formation or merely a response to light signals, we performed a further analysis to see if the expression of these genes reproduced the switch between the on/off states of haustorium formation. In *C. japonica* seedlings, the parasitism-inducing effect of far-red light can be canceled by subsequent exposure to red light (Tada et al. 1996). To ensure that this phenomenon could be reproduced in the in vitro haustorium induction system, *C. campestris* seedlings were exposed to three conditions: (1) Far-red light for 1 h, then 47 h in the dark; (2), Far-red light for 1 h, red light for 1 h, and then 46 h in the dark; and (3) Far-red light for 1 h, red light for 1 h, 2 h in the dark, far-red light for 1 h, and then 43 h in the dark. The rates of haustorium formation were evaluated by microscopic observation.

**Fig. 7:**
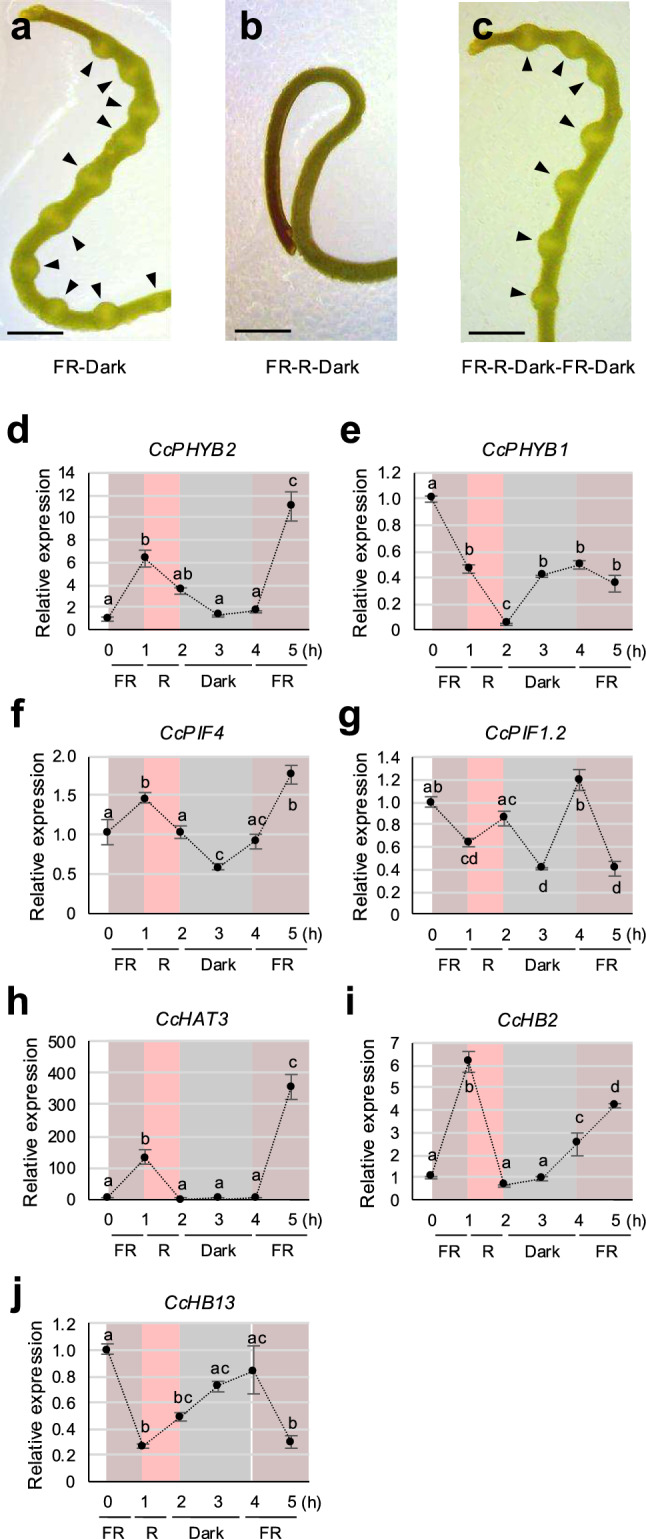
**a-c.** Representative images of a *Cuscuta campestris* seedling incubated in the in vitro haustorium induction system under three different conditions. **a.** Condition 1: 1 h in far-red (FR) light and 47 h in the dark. **b.** Condition 2: 1 h in FR 1 h, 1 h in red (R) light, and 46 h in the dark. **c.** Condition 3: 1 h in FR, 1 h in R, 2 h in the dark, 1 h in FR, and 43 h in the dark. Scale bars: 1 mm. Arrowheads indicate haustoria. **d-j.** qRT-PCR analyses of (**d-e**) *CcPHYB*, (**f-g**) *CcPIFs* and (**h-j**) *CcHD-ZIP I/II* in the in vitro haustorium induction system. *C. campestris* were incubated for 1 h in FR, 1 h in R, 2 h in the dark, and 1 h in FR. Gene expression was assessed 0, 1, 2, 3, 4 and 5 h after the onset of light exposure. Expression of each gene was standardized on *C. campestris TUBULIN* (*CcTUB*, *Co032054.t1*) expression as an internal control and then relative to expression at the start point (0 h). Data shown are ΔCt means ± SE. Different letters indicate significant differences determined by Tukey’s honesty significant difference test at *p* < 0.05

In condition 1, haustoria formed in 90.9% of seedlings (*n* = 11). By contrast, haustorium formation was not observed in condition 2 (0%, *n* = 11). In condition 3, in which seedlings were exposed to, sequentially, far-red, red, and far-red light, haustoria formed in 81.8% of individuals (*n* = 11; Fig. 7a–c). These results suggested far-red light induced haustorium formation in *C. campestris* but subsequent exposure to red light had a dominant effect, canceling the inductive effect of far-red light; however, re-exposing plants to far-red light reversed the negative effect of red light and induced haustorium formation.

We next measured changes in gene expression in seedlings in condition 3. Expression of *CcPHYB2* increased in far-red light; it then clearly declined under red light and remained low in the dark, but increased again upon the second exposure to far-red light (Fig. 7d). *CcPHYB1* expression levels decreased under far-red light, and also under red light, but increased in the dark (Fig. 7e); this pattern was confirmed in constant dark and may be independent of ambient light conditions (Fig. 6b). Expression of *CcPIF4* increased under far-red light, but decreased under red light and in the dark (Fig. 7f); by contrast, *CcPIF1* expression decreased under far-red light, but increased under red light and in the dark (Fig. 7g). These results were consistent with the patterns of *PIF4* and *PIF1* expression observed in Arabidopsis (Lorrain et al. 2008; Shen et al. 2005). Expression of the *CcHD-ZIP I/II* genes *CcHAT3* and *CcHB2* increased under far-red light and decreased under red light (Fig. 7h, i). *CcHB13* expression decreased under far-red light but increased under red light (Fig. 7j). Thus, with the exception of *CcPHYB1*, expression of *CcPHYB2*, *CcPIF1*, *CcPIF4*, *CcHAT3*, *CcHB2* and *CcHB13*, which were identified as DEGs under haustorium-inducing conditions, increased or decreased in a manner matching the switch from far-red to red light exposure. In addition, when conditions were changed a second time from red back to far-red light, gene expression again responded accordingly. These results demonstrated the strong correspondence between gene expression and the initiation of haustorium formation, highlighting the unique light-dependent behavior of *PHYB* in *C. campestris*.

## Discussion

### The initial molecular response to specific light signals induces haustorium formation in *C. campestris*

*Cuscuta* parasitism requires exposure to specific light signals, such as blue or far-red light, but how the plants use such information to induce haustorium formation has not previously been elucidated. We determined that 3 to 4 h was a sufficient period of light exposure to induce haustorium formation and designated this time as the light-regulated haustorium “induction stage” (Fig. 1). Although this stage was not marked by any discernible morphological changes, it was an important step that governed the transition to haustorium formation. The commitment to form a haustorium could, however, be canceled by exposure to red light. Our research revealed a unique set of genes that were activated in *C. campestris* prior to the onset of haustorium formation; these genes, *CcPHYB2*, *CcPIF1*, *CcPIF4*, *CcHAT3*, *CcHB2* and *CcHB13*, were identified through their responses to the light signal that induced haustorium formation but preceded visible morphological change. In addition to the genes mentioned above, our transcriptomic analysis identified Arabidopsis orthologs of a blue light receptor, genes involved in auxin biosynthesis, and ethylene signaling. These include *CRY1* (*Cc005583.t1*), *YUC10* (*Cc001518.t1, Cc044976.t1*), multiple *SAUR-like* genes (*Cc001777.t1, Cc001779.t1, Cc001781.t1, Cc023889.t1, Cc023890.t1, Cc023891.t1, Cc023892.t1*), as well as *ERF5* (*Cc002540.t1*), *ETR2* (*Cc004838.t1, Cc031639.t1*), and *EIN3* (*Cc016776.t1*), all of which were differentially expressed during the haustorium induction stage (Table S1). These findings suggest that, in addition to the PHY-regulating light signaling, blue light signaling and a possible interplay between light and hormonal signaling pathways may play a role in haustorium induction in *C. campestris*. Especially, as only the combination of blue light and red light, but not far-red light and red light, had inhibitory effect of haustorium formation (Yokoyama et al. 2023), it is clear the blue and far-red light pathways do not completely overlap.

Although further investigation is needed to determine a regulatory mechanism in the haustorium induction process in *C. campestris*, one hypothetical mechanism is the contribution of PIF4 to the unique light-dependent parasitic behavior. In Arabidopsis, AtPIF4 is a key bHLH transcription factor that functions downstream of AtPHYB and acts as an integrator of light and phytohormonal signals. (Pham et al. 2018). The stability of the AtPIF4 protein is regulated by AtPHYB through light-dependent proteasome-mediated degradation, and AtPIF4 directly regulates the expression of genes involved in auxin biosynthesis and transport, including *YUCCA*, *SAUR* and *IAA* family members, contributing to the shade avoidance response (Goyal et al. 2016; Nozue et al. 2011; Oh et al. 2014). Moreover, extensive research has shown that PIF and HD-ZIP I/II proteins work together in light-signaling pathways in Arabidopsis. HD-ZIP II is particularly responsive to light stress and canopy shade (Sharif et al. 2021). AtPIF4 and AtPIF5 are known to directly interact with ARABIDOPSIS THALIANA HOMEOBOX 2 (ATHB2, a member of the HD-ZIP II family) (Hornitschek et al. 2012; Leivar et al. 2012; Lorrain et al. 2008) and regulate shade-induced growth responses by controlling auxin fluxes (He et al. 2020).

While auxin signaling plays an important role in haustorium initiation, different genes acting downstream of light perception are responsible for the various stages of haustorium development, from the initial, invisible responses that trigger the switch to formation to the later, more obvious “swelling stage”, which is the first visual sign of haustorium formation. The swelling stage occurs when flat epidermal tissue bumps appear 1 to 2 d after light irradiation; it is marked by three genes, *Cc008373.t1*, *Cc009295.t1*, and *Cc015960.t1* (Bawin et al. 2022). Other putative key players include *CcHB7* (*Cc014209.t1*), *CcPMEI* (*Cc038093.t1*), *CcERF1* (*Cc002541.t1*), and *CcLBD25* (*Cc019141.t1*), which all show clear variation in local expression as the haustorium invades host tissue (Jhu et al. 2021, 2022). These genes, however, were not among the DEGs identified in the light-regulated induction stage (Table S1). In root parasitic plants, auxin-responsive genes, such as AUXIN RESPONSE FACTORS (ARFs) and LBDs, play significant roles in haustorium formation (Ichihashi et al. 2020; Yoshida et al. 2019). Auxin-responsive genes, such as *CcLBD25*, which acts during the later stages of haustorium formation, were not among the DEGs identified in our analysis (Table S1).

### Diversification of PHYB genes in *C. campestris* and their potential roles in haustorium initiation

Although it has long been hypothesized that phytochrome is involved in *Cuscuta* parasitism (Bawin and Krause 2024), previous studies have not specifically focused on the gene expression of phytochrome and phytochrome-related genes in *Cuscuta*. We identified putative phytochromes in *Cuscuta* and performed a phylogenetic analysis. This revealed that *Cuscuta* species possessed two phylogenetically distinct PHYBs, *Cuscuta* PHYB1 and *Cuscuta* PHYB2 (Fig. 3b). Phylogenetic analysis showed that, although *Cuscuta* PHYB1 was closely related to *Ipomoea* PHYB, *Cuscuta* PHYB2 formed a separate clade distant from orthologs from related species (Fig. 3b). This clade was present in all *Cuscuta* genomes sequenced to date, suggesting that PHYB diversification occurred during the evolution of the genus.

Many post-translational light-responsive behaviors of phytochrome are well characterized, including light-dependent nuclear translocation, photo-reversible conformational changes, and phytochrome-regulated gene expression by phytochrome (Cheng et al. 2021). Phytochrome gene expression, however, is independent of the light spectrum in the environment (Dehesh et al. 1991; Sharrock and Quail 1989). We found *CcPHYB1* was expressed constitutively, regardless of the light conditions, which was consistent with PHYB expression in Arabidopsis and rice (*Oryza sativa*) (Dehesh et al. 1991; Sharrock and Quail 1989). By contrast, expression of *CcPHYB2* was light-dependent, with higher levels observed under blue and far-red light conditions. PHYB diversification and spatial–temporal differences in expression have been observed previously in a range of plant species, including poplar (*Populus trichocarpa*), corn (*Zea mays*), and tomato (*S. lycopersicum*), suggesting functional diversification within the PHYB family (Hauser et al. 1997; Karve et al. 2012; Sheehan et al. 2007). Thus, *Cuscuta* PHYB1 and PHYB2 may be subfunctionalized. In *C. campestris*, CcPHYB1 and other constitutively expressed CcPHYs may play a role in monitoring the ambient light environment during the search for a host, while CcPHYB2, which was expressed specifically during the light-regulated induction stage, may regulate the switch to haustorium formation. Although further research is required to elucidate the precise function(s) of the different PHYBs in haustorium formation, our study emphasizes the likelihood that these genes, which are part of the light signal transduction machinery in *C. campestris*, are involved in the initial steps of light-regulated haustorium formation.

## Supplementary Information

Below is the link to the electronic supplementary material.

Supplementary file1 (PDF 403 KB)

Supplementary file2 (XLSX 20 KB)

Supplementary file3 (XLSX 14 KB)

Supplementary file4 (XLSX 10 KB)

## Data Availability

The data that support the findings of this study are available from the corresponding author upon reasonable request.
