# Supplementary material for: A unique phytochrome B gene in Cuscuta campestris and its responses during the initial stage of haustorium formation
Source: J Plant Res. 2025 Jun 24;138(5):857–72. doi: 10.1007/s10265-025-01653-5 (PMC12441084; doi:10.1007/s10265-025-01653-5)
Supplement: Supplementary file 1 — Supplementary file1 (PDF 403 KB) [file 10265_2025_1653_MOESM1_ESM.pdf]

Article Title:

A unique phytochrome B gene in *Cuscuta campestris* and its responses during the initial stage of haustorium formation

Journal name:
Journal of Plant Research

Author:
Toshiya Yokoyama[1], Mariko Asaoka[1,*], Kazuhiko Nishitani[1]

Affiliation:
[1] Department of Science, Faculty of Science, Kanagawa University, Yokohama, Kanagawa, Japan

*Corresponding author for Contact:
Mariko Asaoka
asaoka@kanagawa-u.ac.jp

Supplementary Figure 1

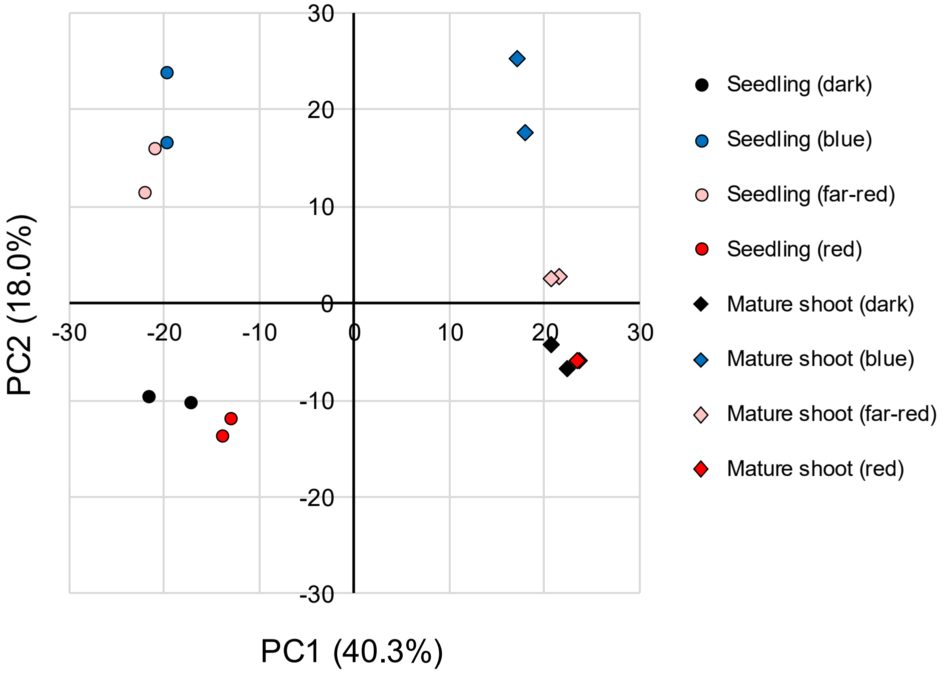

Figure S1. Principal Component Analysis (PCA) of gene expression profiles in the whole RNA-seq data set.

Supplementary Figure 2

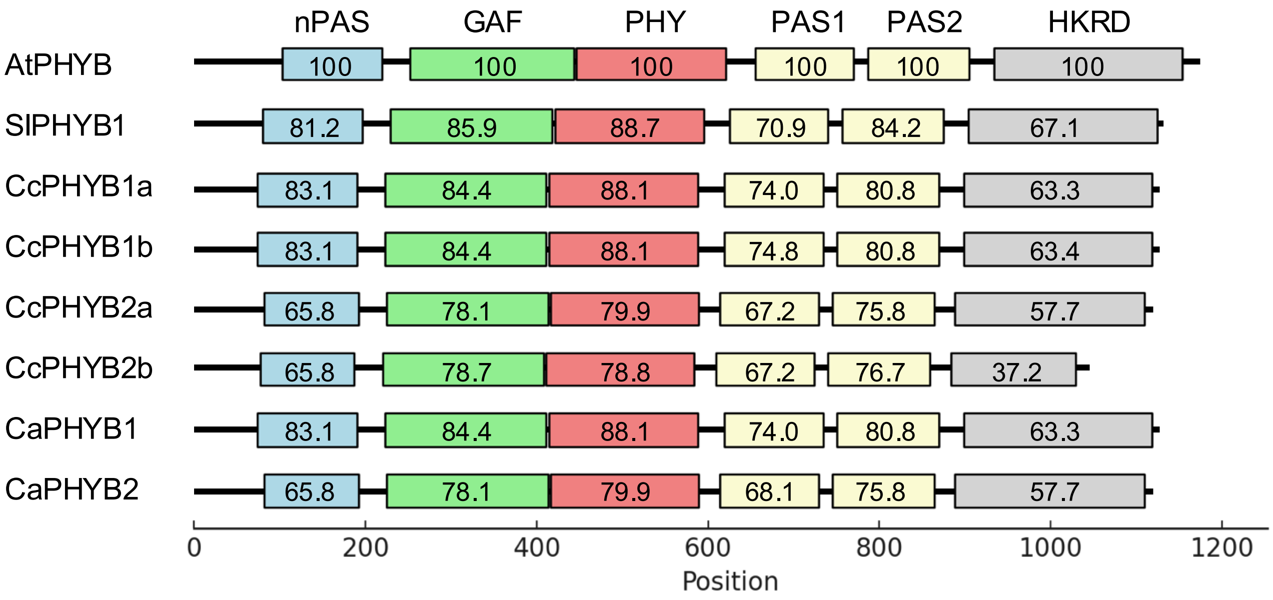

Figure S2. Conserved domains in phytochrome B protein sequences. Conserved domains in the *Arabidopsis thaliana* (At), *Solanum lycopersicum* (Sl), *Cuscuta campestris* (Cc) *Cuscuta australis* (Ca) sequences were identified using the InterPro website (https://www.ebi.ac.uk/interpro/). The numbers within each domain represent the percentage of identity of that domain compared to AtPHYB.

Supplementary Figure 3

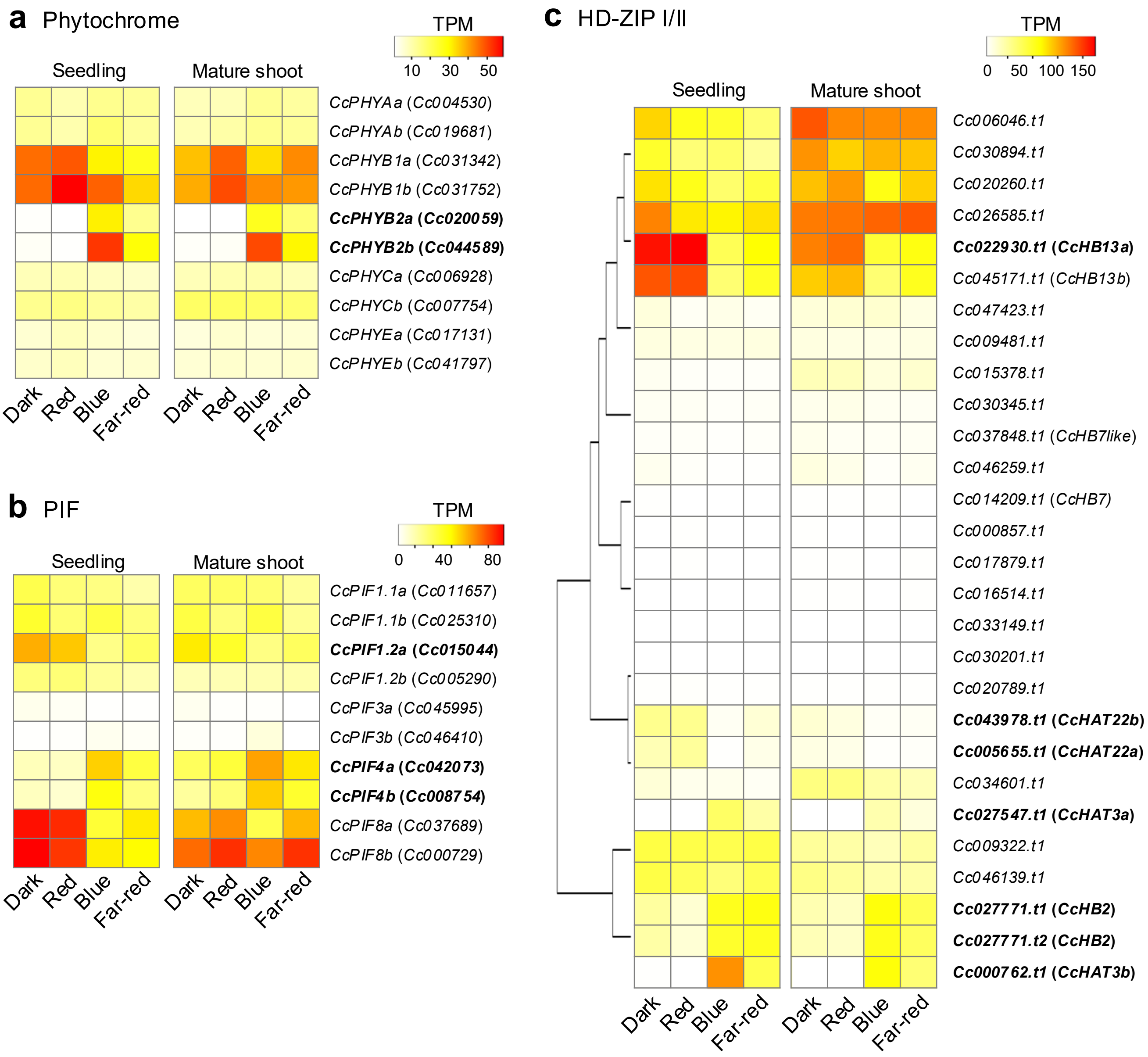

Figure S3. Heatmap of transcripts per million (TPM) showing normalized results from the RNA-seq analysis of plants incubated in the *in vitro* haustorium induction system. Heatmap shows gene expression profiles (TPM) of *CcPHYs, CcPIFs* and *CcHD-ZIP I/II* according to the color scale. Genes in the GS3 (Fig. 1c), *i.e.,* differentially expressed genes (DEGs) common to both seedlings and mature shoots under blue and far-red light conditions, are shown in bold.
